# Investigation of the relationship between *Fusobacterium nucleatum* presence and inflammatory mediator expression in Kazakhstani patients with colorectal cancer

**DOI:** 10.3389/fmicb.2025.1699745

**Published:** 2025-11-05

**Authors:** Gulmira Kulmambetova, Botakoz Kurentay, Alua Gusmaulemova, Dina Bayanbek, Meiram Mamlin, Saule Khamzina, Sanzhar Shalekenov, Arman Kozhakhmetov

**Affiliations:** ^1^Department of Genomics, National Center for Biotechnology, Astana, Kazakhstan; ^2^Multidisciplinary Surgery Center, National Scientific Oncology Center, Astana, Kazakhstan; ^3^Department of Surgery, Nazarbayev University School of Medicine, Astana, Kazakhstan

**Keywords:** colorectal cancer, ddPCR, expression, *Fusobacterium nucleatum*, interleukins, Kazakhstan

## Abstract

**Objectives:**

*Fusobacterium nucleatum* has been recognized as a critical microorganism contributing to the development and progression of colorectal cancer (CRC). However, the role of *F. nucleatum* in colorectal cancer, including its effects on immune factors and the tumor microenvironment, remains unclear. This study aimed to explore the relationship between the presence of *F. nucleatum* and the expression of inflammation-related genes (IL6, IL1B, IL10, IL17, TNF) in tumor and matched normal tissue of Kazakhstani CRC patients.

**Methods:**

The abundance of *F. nucleatum* was detected in 113 paired tumor and normal tissue specimens by quantitative PCR technology (qPCR). The interleukin expression levels of cytokines (IL-6, IL-1β, IL-10, IL-17, TNF-*α*) were examined by TagMan droplet digital PCR technology (ddPCR). Finally, we investigated the potential associations between the molecular and clinicopathological characteristics of the samples and the abundance of *F. nucleatum*.

**Results:**

The relative abundance of *Fusobacterium nucleatum* was significantly higher in cancerous tissues compared to normal tissues. Moreover, the expression levels of IL-6 and IL-1β were significantly elevated in the cancer group. A strong correlation was also found between high levels of *Fusobacterium nucleatum* and increased expression of IL-17. In addition, increased levels of *Fusobacterium nucleatum* were significantly associated with histological grade II and III colorectal cancer tissues, as well as with certain clinical characteristics, including microsatellite instability (MSI), patient nationality, and processed meat intake (*p* < 0.05).

**Conclusion:**

Our findings highlight the significance of *Fusobacterium nucleatum* and the alterations in gene expression associated with colorectal cancer. Investigating the microbial landscape and gene expression patterns in CRC patients could offer a valuable approach for enhancing screening techniques and developing effective therapeutic strategies.

## 1 Introduction

Colorectal cancer (CRC) remains a leading cause of cancer incidence and death worldwide, with increasing incidence rates reported across various populations. As reported by the World Health Organization (WHO), colorectal cancer ranks as the third most frequently diagnosed cancer globally, representing about 10% of all cancer cases, and stands as the second major contributor to cancer mortality worldwide (WHO, n.d.). While genetic and environmental factors have long been recognized as central contributors to colorectal carcinogenesis, mounting evidence highlights the critical role of the gut microbiota in modulating cancer risk and progression. Among the numerous microbial species implicated, *Fusobacterium nucleatum* has emerged as a key player in CRC development due to its frequent enrichment in tumor tissues compared to adjacent normal mucosa.

*Fusobacterium nucleatum* is a gram-negative anaerobic bacterium commonly residing in the oral cavity, but it has also been detected at elevated levels in colorectal tumors. Its role in colorectal cancer is thought to occur through multiple mechanisms, most notably its capacity to trigger inflammation, a recognized hallmark of cancer.

An overabundance of *F. nucleatum* in CRC tissues has also been reported (Han, 2015; Kostic et al., 2012; Castellarin et al., 2012). *F. nucleatum* adheres to colorectal tissues through at least two distinct mechanisms: the FadA pathway and the fusobacterial Gal-GalNAc–binding lectin (Fap2) pathway. FadA, a surface-expressed adhesin, is produced by *F. nucleatum* (Rubinstein et al., 2013). *F. nucleatum* adheres to and invades colorectal cells through its FadA, which binds to E-cadherin (Rubinstein et al., 2013). Within the Fap2 pathway, fusobacterial Fap2 recognizes the host polysaccharide Gal-GalNAc, which is overexpressed in colorectal cancer (Abed et al., 2016). It is hypothesized that fusobacteria colonizing CRC may originate from the oral cavity, as they are core members of the human oral microbiome, are rarely detected in the gut, and transient bacteremia frequently occurs during periodontal disease (Pignatelli et al., 2023; Chen et al., 2022). In view of previous findings, the abundance of *F. nucleatum* in CRC may serve as a prognostic marker.

Chronic inflammation creates a tumor-promoting microenvironment characterized by immune cell infiltration, the release of cytokines, and the activation of pro-oncogenic signaling pathways. Notably, *F. nucleatum* has been shown to modulate host immune responses by interacting with pattern recognition receptors and influencing the expression of key inflammatory mediators such as interleukins IL-6, IL-1β, IL-17, IL-10, and tumor necrosis factor-alpha (TNF-*α*). Understanding the interplay between *F. nucleatum* and inflammatory signaling is essential for elucidating its role in CRC pathogenesis.

Investigating the association between bacterial abundance and the expression profiles of inflammatory cytokines in colorectal tissues may provide insights into microbial contributions to tumor development and progression. Few studies have examined how *F. nucleatum* interacts with and regulates inflammatory genes in CRC (Brennan et al., 2021; Engevik et al., 2021). In this study, we employed digital PCR, a method with a sensitivity approximately 1,000-fold greater than that of quantitative real-time PCR.

This study investigates the association between *Fusobacterium nucleatum* presence and the expression of inflammation-related genes (IL6, IL1B, IL10, IL17, TNF) in tumor and matched normal tissues from Kazakhstani patients with colorectal cancer.

## 2 Materials and methods

### 2.1 Sample preparation

A total of 113 paired colorectal adenocarcinoma and matched non-tumor tissue biopsies were obtained from the National Research Oncology Center between 2022 and 2024 (Supplementary Table 1).

Patients with a history of other malignancies, prior preoperative radiotherapy or chemotherapy, or evidence of distant metastases were excluded. Biopsies were collected from carcinoma tissues (CTs) and distant normal tissues (NTs, located 10 cm beyond the tumor margin) of CRC patients. A total of 226 samples were stored in 20% sucrose solution. Nucleic acids for qPCR and ddPCR analyses were extracted from colon tissue samples, while the remaining material was stored at −80 °C until further use. All 113 patients were analyzed by qPCR.

The study was conducted in accordance with the Declaration of Helsinki and approved by the Local Ethics Committee of the National Center for Biotechnology, Ministry of Health of the Republic of Kazakhstan (Protocol No. 1, dated 01 April 2022). All experimental procedures were performed in accordance with relevant guidelines and regulations, and written informed consent was obtained from all participants.

### 2.2 Nucleic acid extraction and reverse transcription

Genomic DNA and total RNA were isolated from tumor and paired normal mucosa using the QIAamp DNA Mini Kit and RNeasy Plus Mini Kit (QIAGEN, Germany; Cat. Nos. 51,304 and 74,134), respectively, following the manufacturer’s protocols. The concentration and purity of nucleic acid extracts were assessed using a NanoDrop 1,000 spectrophotometer (Thermo Fisher Scientific, USA) at OD 260/280 nm. Reverse transcription was performed with the BioMaster RNAscribe RT Plus kit (Biolabmix, Cat. No: R02-100). Both genomic DNA and synthesized cDNA were stored at −20 °C until further use.

### 2.3 Relative quantification of *F. nucleatum* by qPCR

The abundance of *F. nucleatum* in cancerous and matched normal tissues was determined by targeting the *nusG* gene using SYBR Green–based real-time PCR on a CFX384 Touch Real-Time PCR Detection System (Bio-Rad, USA). The human *solute carrier organic anion transporter family member 2A1* (*SLCO2A1*) gene served as an internal reference for normalization of quantification cycle (Cq) values, as previously described (Mima et al., 2016). *SLCO2A1* was validated as a suitable reference gene (Supplementary Table 2), as no significant differences in its Ct values were observed between tumor and matched normal tissues or among tumor stages (Supplementary Figure 1). Each 10 μL PCR reaction contained 20 ng of genomic DNA, 10 μM of each primer, 5 μL of SYBR Green Master Mix (Biolabmix, Russia), and nuclease-free water.

qPCR was performed with an initial denaturation at 95 °C for 15 min, followed by 40 cycles of denaturation at 95 °C for 15 s and annealing/extension at 62 °C for 30 s. Each sample was analyzed in duplicate within the same run, and the mean values were used for statistical analysis. Negative controls included all reaction components except bacterial genomic DNA, while *F. nucleatum* (accession no. JARWBA000000000) served as a positive control. Relative abundance was calculated using the 2^−ΔΔCq method, where ΔCq represents the difference between the mean Cq values of *F. nucleatum* and the reference gene, and ΔΔCq was obtained by subtracting ΔCq of normal tissue from ΔCq of tumor tissue (Livak and Schmittgen, 2001).

### 2.4 Expression of inflammatory and anti-inflammatory genes

In this study, relative quantification of interleukin (*IL-6, IL-10, IL-1β, IL-17,* and *TNF-α*) gene expression was performed using digital droplet PCR (ddPCR) with TaqMan primer/probe assays. Reactions were carried out on a QX200 Droplet Digital PCR System (Bio-Rad, USA) according to the manufacturer’s instructions. Each 20 μL reaction mixture contained 10 μL of ddPCR Supermix for Probes (no dUTP) (Bio-Rad, USA), 1 μL of target probe (FAM, 20X), 1 μL of reference probe for *GAPDH* (VIC), 3 μL of nuclease-free water, and 5 μL of cDNA. Droplets were generated in a DG8 cartridge by adding the PCR mix to the middle wells and 70 μL of droplet generation oil to the bottom wells. No-template controls were included in all runs. The thermal cycling protocol consisted of an initial denaturation at 95 °C for 3 min, followed by 40 cycles of 95 °C for 30 s and 60 °C for 60 s (adjusted depending on primer annealing temperature). *GAPDH* was used as an internal reference gene, and cDNA levels were calculated using the relative expression method described above. Data acquisition and analysis were performed with QuantaSoft software (Bio-Rad, USA).

### 2.5 Statistical analyses

Statistical analyses were performed using R software (version 4.4.2^1^) within the RStudio environment (version 2022.02.2). A two-tailed *p* value < 0.05 was considered statistically significant. Paired-samples *t*-tests were applied to compare the relative abundance of *F. nucleatum* and the expression levels of proinflammatory genes between tumor tissues and matched adjacent normal mucosa. Associations between *F. nucleatum* status and clinicopathological or molecular characteristics were evaluated using Fisher’s exact test or the chi-squared test (χ^2^), as appropriate. Multivariate logistic regression analysis was used to calculate odds ratios (ORs) and 95% confidence intervals (CIs).

## 3 Results

### 3.1 Clinicopathological characteristics of CRC patients

The histopathological and demographic characteristics of the patients are summarized in Table 1. The cohort included 49 men and 62 women, with a mean age of 61.7 years (SD ± 10.9). Most CRC cases were classified as grade III (poorly differentiated, 44.2%), followed by grade II (moderately differentiated, 41.6%), grade I (well-differentiated, 11.5%), and grade X (undetermined, 2.7%). Tumor localization, based on gastroenterological and pathological examinations, was predominantly in the sigmoid colon (42.5%) and rectosigmoid region (18.6%), with additional cases in the ascending colon (15%) and cecum (9.7%). All 113 patients were diagnosed with colorectal adenocarcinoma.

**Table 1:** Clinicopathological characteristics and molecular profiles in patients with high versus low *F. nucleatum* abundance.

Patient characteristics	*F. nucleatum* abundance in CRC tissue		
	*Fn* – low	*Fn –* high	*p*
Age in years			
Mean ± SD	64.00 ± 10.36	62.50 ± 11.47	0.12
Sex			
Male	25	24	1.00
Female	32	30	
BMI			
Mean ± SD	26.23 ± 4.54	27.51 ± 4.89	0.16
Tumor location			
Right	13	21	0.09
Left	44	33	
Tumor size			
Mean ± SD (сm)	4.86 ± 3.06	4.48 ± 1.61	0.41
CEA			
Mean ± SD	18.06 ± 37.03	91.77 ± 402.57	0.39
pT			
pTis	0	2	0.37
1	1	1	
2	7	8	
3	35	36	
4	14	7	
pN			
0	32	29	0.85
1 or 2	25	25	
pStage			
I	7	6	0.93
II	25	21	
III	25	24	
IV	0	1	
Tumor differentiation			
Well (G1)	0	1	0.57
Moderate (G2)	27	22	
Poor (G3)	30	31	
MSI			
Low	28	29	**0.03**
High	0	6	
Alcohol			
None drinker	54	52	0.62
Social drinker	3	1	
Smoking status			
Non-smoker	48	46	1.00
Smoker	9	8	
Diabetes			
Yes	17	8	0.07
No	40	46	
Hypertension			
Yes	37	21	**0.01**
No	20	33	
Nationality			
Asians	46	32	**0.01**
Europeans	10	22	
Red meat consumption (g/day)			
Mean ± SD	176.73 ± 134.57	170.8 ± 96.13	0.85
Processed meat consumption (g/day)			
Mean ± SD	7.73 ± 15.71	41.17 ± 52.71	**0.0009**
Total meat consumption (g/day)			
Mean ± SD	238.5 ± 215.49	263 ± 191.07	0.61

Bold indicates significant values.

### 3.2 Quantification of *Fusobacterium nucleatum*

**Figure 1:**
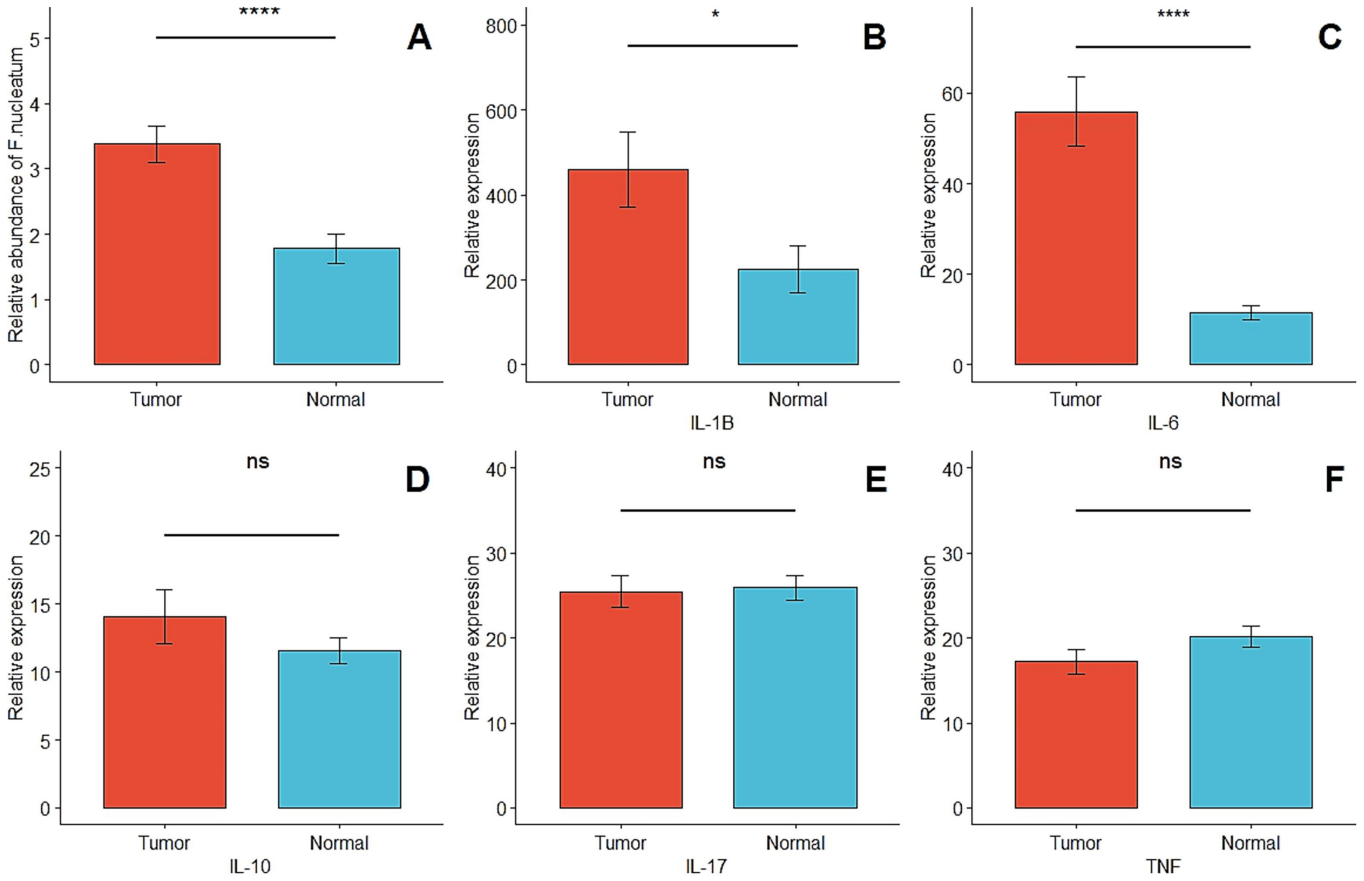
**(A)** Comparison of the presence of *F. nucleatum* in tumor and matched normal tissues (*p* = 0.0005). **(B–F)** Relative quantification of inflammatory and anti-inflammatory genes in tumor and matched normal tissues (*p* < 0.05^*^ and *p* > 0.05^ns^).

In this study, CRC-associated *Fusobacterium nucleatum* was quantified in colorectal carcinoma tissues and matched normal mucosal samples using relative quantification real-time PCR. The mean abundance of *F. nucleatum* was significantly higher in tumor tissues compared with normal tissues (CRC vs. normal: 3.2 ± 3.06 vs. 1.7 ± 2.23, *n* = 113, *p* = 0.0005, paired *t*-test) (Figure 1A). *F. nucleatum* was detected in 35.4% of cancer tissues and 19.5% of non-tumor tissues. The median relative abundance in tumor tissue was used to define an optimal cutoff for categorizing *F. nucleatum*-positive CRCs into low- and high-level groups. Of the 40 CRC cases with detectable *F. nucleatum*, 36 were classified as high-level and 4 as low-level. Patients in the high-level group had no prior history of cancer, with a mean age of 59.9 years (SD ± 11.5) and a mean tumor size of 4.47 cm (SD ± 1.61). Statistical analysis revealed no significant correlations (*p* > 0.05) between *F. nucleatum* abundance and the examined clinicopathological markers.

### 3.3 Inflammatory and anti-inflammatory gene expression

In this study, the expression levels of *IL6, IL10, IL1B, IL17,* and *TNF-α* were quantified using ddPCR and analyzed as relative expression. The median copies/μL of cDNA were used to classify each gene’s expression in CRC samples into low- and high-expression groups. *IL6* and *IL1B* were significantly upregulated in tumor tissues compared with matched normal mucosa (*p* < 0.05). No significant differences were observed for *IL10, IL17,* or *TNF-α* between tumor and normal tissues (*p* > 0.05) (Figures 1B–F). The relative abundance of *Fusobacterium nucleatum* and the expression levels of cytokine genes (IL-1β, IL-6, IL-10, IL-17, TNF) were measured in tumor and adjacent normal tissues to assess their differential expression and potential associations. This analysis was conducted to investigate the potential link between *Fusobacterium nucleatum* colonization and inflammatory cytokine activation in the colorectal tumor microenvironment.

### 3.4 Clinicopathological and molecular association of *F. nucleatum* in CRC

The clinicopathological and molecular characteristics of CRC patients according to *Fusobacterium nucleatum* status (high vs. low expression) are summarized in Tables 1, 2. Patients were divided into two groups based on the median *F. nucleatum* Ct value in tumor tissue DNA (8.6 Ct). Those with Ct values below 8.6 were classified as the *F. nucleatum*-high group, and those with Ct values above 8.6 as the *F. nucleatum*-low group. This stratification allowed comparison of the frequency of high- versus low-level *F. nucleatum* and assessment of its association with clinicopathological features.

**Table 2:** Association between *F. nucleatum* status and molecular characteristics.

No. (%)					
Amount of *F. nucleatum* in colorectal carcinoma tissue					
Gene		All patients (*n* = 102)	Low (*n* = 67)	High (*n* = 35)	*p*-value
IL6	High	52	40 (59.70)	12 (34.29)	**0.02**
	Low	50	27 (40.30)	23 (65.71)	
IL1B	High	50	36 (53.73)	14 (40.00)	0.21
	Low	52	31 (46.27)	21 (60.00)	
IL10	High	51	30 (44.78)	21 (60.00)	0.21
	Low	51	37 (55.22)	14 (40.00)	
IL17	High	55	42 (62.69)	13 (37.14)	**0.02**
	Low	47	25 (37.31)	22 (62.86)	
TNF	High	50	30 (44.78)	20 (57.14)	0.29
	Low	52	37 (55.22)	15 (42.86)	

Bold indicates significant values.

Fisher’s exact test revealed a significant correlation between high levels of *Fusobacterium nucleatum* and microsatellite instability (MSI) status in CRC tissues (*p* = 0.01), with higher *F. nucleatum* DNA levels observed in MSI-high tumors compared to MSI-low tumors. Additionally, patients with hypertension exhibited a significant association with low-level *F. nucleatum* (*p* = 0.01). Among European patients with invasion-positive tumors, *F. nucleatum* DNA levels were higher compared to Asian invasion-positive patients (*p* = 0.01). High processed meat consumption was also significantly associated with elevated *F. nucleatum* levels (*p* = 0.0009). No significant correlations were observed between *F. nucleatum* abundance and other clinicopathological variables (*p* > 0.05).

In multivariable logistic regression analysis, hypertension (OR = 2.57; 95% CI: 1.15–5.85; *p* = 0.02), tumor location (OR = 2.82; 95% CI: 1.15–7.26; *p* = 0.03), and pT stage (OR = 1.85; 95% CI: 1.03–3.56; *p* = 0.04) were significantly associated with increased odds of high *F. nucleatum* levels in tumor tissues, whereas pN stage showed no statistically significant association (OR = 0.69; 95% CI: 0.36–1.29; *p* = 0.25).

**Figure 2:**
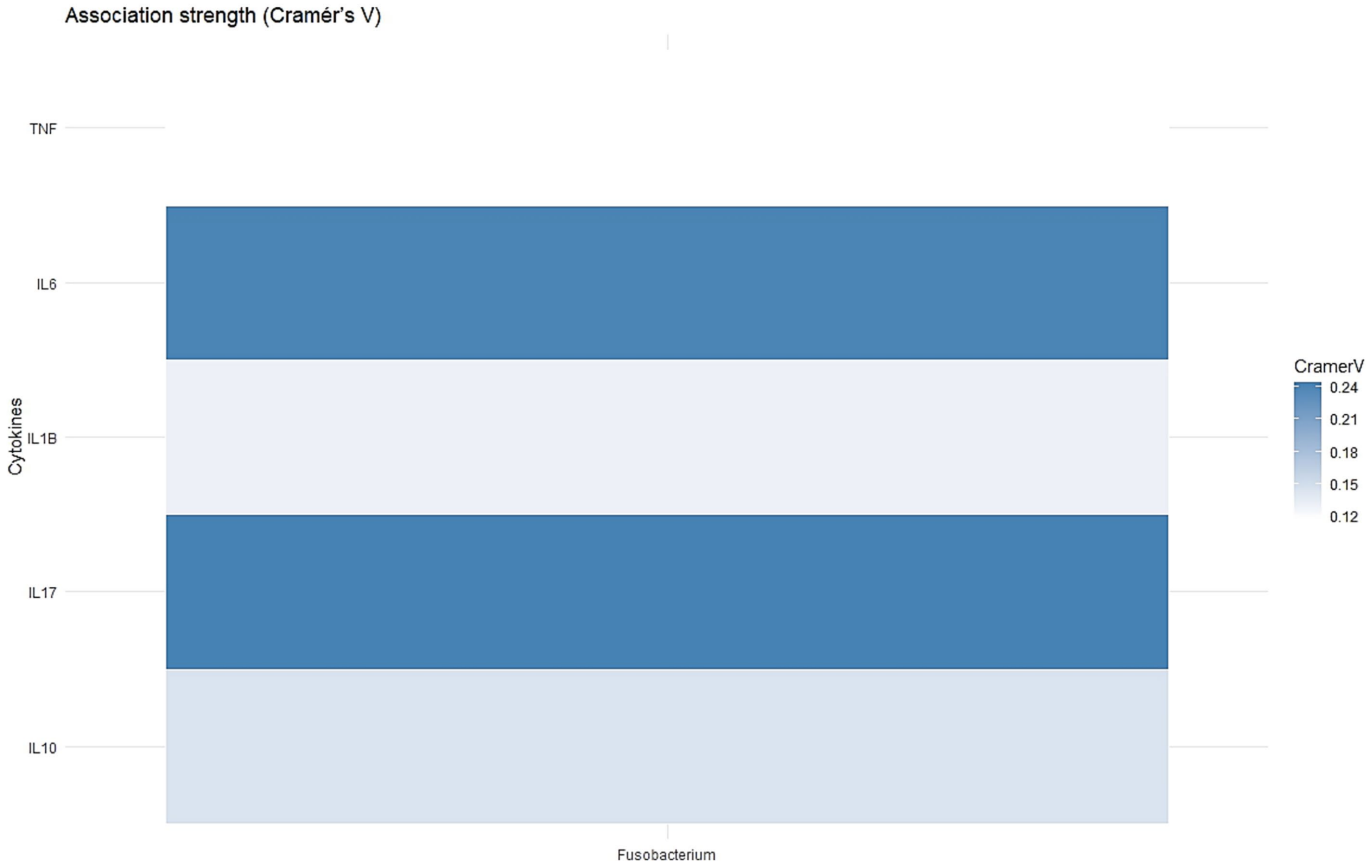
Associations between *Fusobacterium* abundance and cytokine gene expression in CRC.

CRC patients were divided into three groups based on tertiles of *Fusobacterium nucleatum* Ct values in tumor DNA, with the 33rd percentile corresponding to a Ct of 6.8. Patients with Ct values below 6.8 were classified as the *F. nucleatum*-high group, and those above 6.8 as the *F. nucleatum*-low group, enabling comparison of *F. nucleatum* abundance with molecular features. Significant correlations were observed between higher *F. nucleatum* levels and elevated expression of *IL6* (*p* = 0.02) and *IL17* (*p* = 0.02) (Figure 2). No significant associations were observed between *F. nucleatum* abundance and the expression of the other examined genes (*p* > 0.05). The strength of association between *Fusobacterium nucleatum* abundance and cytokine gene expression was evaluated using Cramér’s V to determine whether higher bacterial loads are linked to alterations in the inflammatory cytokine profile of colorectal cancer tissues.

In multivariable logistic regression analysis, *IL17* expression was significantly associated with increased odds of high *Fusobacterium nucleatum* levels in tumor tissues (OR = 3.23; 95% CI: 1.22–9.11; *p* = 0.02). *IL10* showed a borderline inverse association with *F. nucleatum* abundance (OR = 0.37; 95% CI: 0.12–1.04; *p* = 0.07). No significant associations were observed for *IL1B, TNF,* or *IL6*.

### 3.5 *F. nucleatum* abundance in CRC tissues

**Figure 3:**
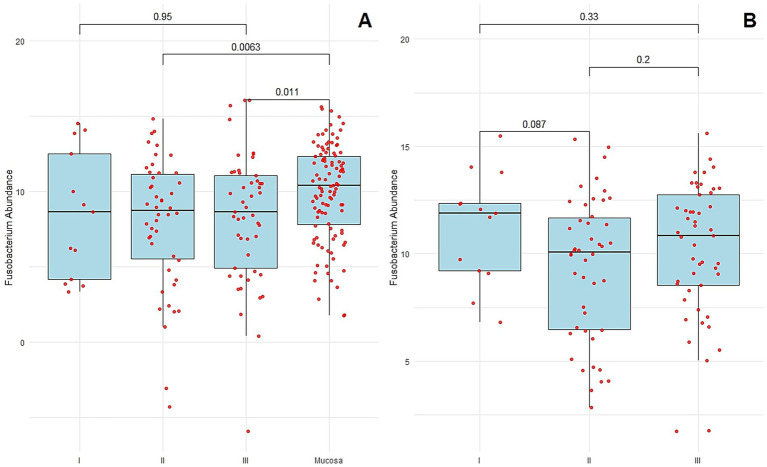
**(A)** *F. nucleatum* abundance in the normal distant tissues of mucosa and colorectal cancer tissues of each stage. **(B)** *F. nucleatum* abundance in the normal distant tissues of mucosa of each stage. Each sample is indicated by a *red point*. The *box plots* show the median with interquartile range (25th percentile and 75th percentile).

*Fusobacterium nucleatum* was detected in 19.5% (22/113) of normal distant mucosal tissues and 35.4% (40/113) of CRC tissue samples. The abundance of *F. nucleatum* was significantly higher in CRC tissues compared to matched normal mucosa (*p* = 0.0005, paired *t*-test) (Figure 1A). The median Ct value of *F. nucleatum* in tumor tissue was 8.5, compared with 10.2 in normal distant mucosa (*p* = 0.001, Wilcoxon matched-pairs signed rank test). No statistically significant difference in *F. nucleatum* abundance was observed between normal mucosa and stage I–III CRC tissues overall (*p* = 0.87, Kruskal–Wallis test). However, significant associations were observed when comparing normal mucosa to stage II and stage III CRC tissues specifically (median Ct 8.7 vs. 10.2, *p* = 0.006; median Ct 8.6 vs. 10.2, *p* = 0.01, respectively) (Figure 3A).

*F. nucleatum* abundance in normal-distant mucosa to CRC

No statistically significant differences in *Fusobacterium nucleatum* abundance were observed among normal distant mucosa from CRC patients at different stages (*p* = 0.16, Kruskal–Wallis test). As shown in Figure 3B, *F. nucleatum* abundance in normal distant mucosa was not significantly higher in stage III CRC patients (median Ct = 10.8) compared with stage I (median Ct = 11.9, *p* = 0.33), stage II (median Ct = 10.08, *p* = 0.20), or between stages I and II (*p* = 0.08).

### 3.6 Correlation between *F. nucleatum* levels and clinical outcomes

**Figure 4:**
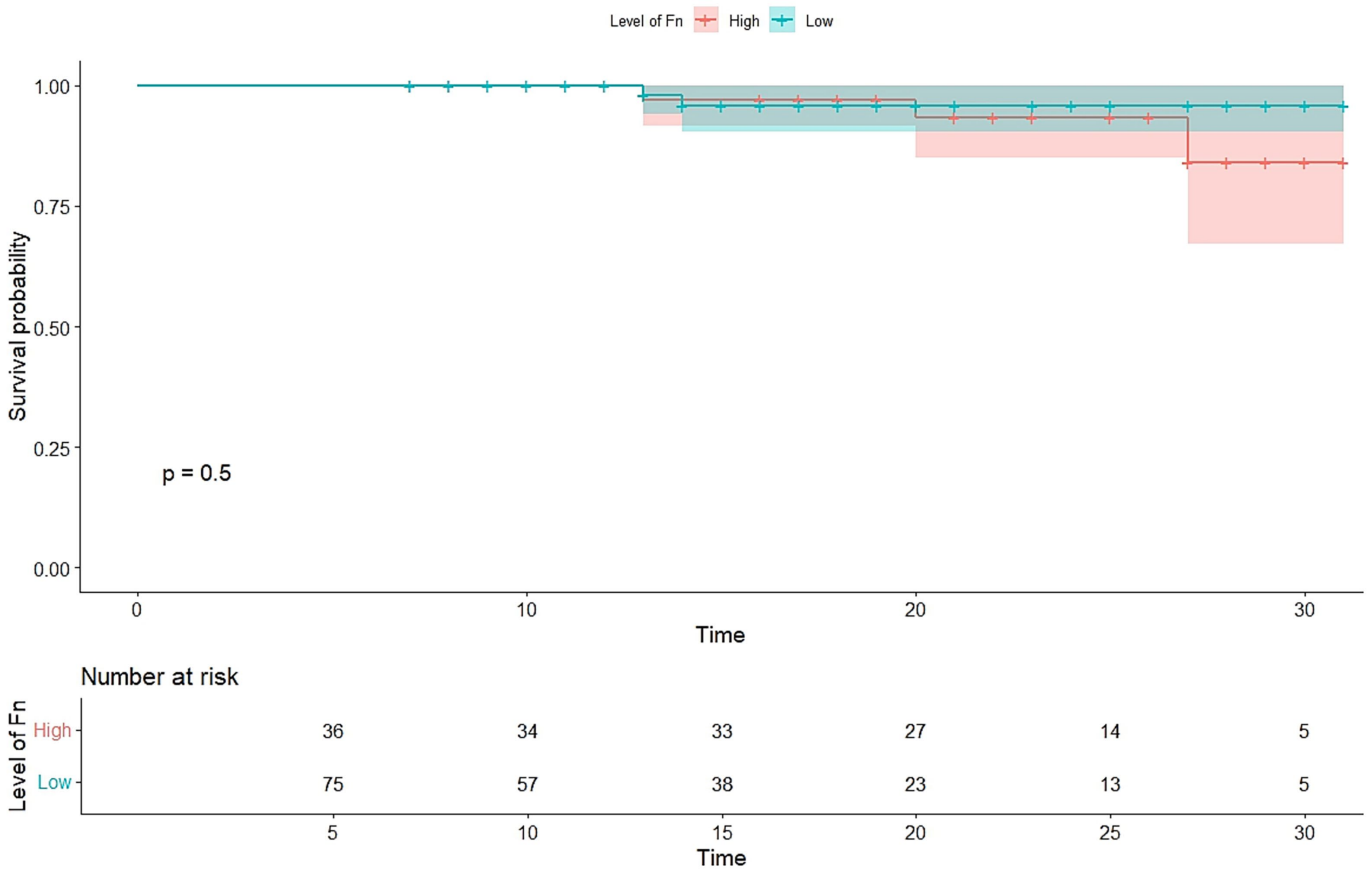
Kaplan–Meier plots of overall survival. Overall survival in patients with colorectal cancer of all stages. The patients were classified into two groups according to the abundance *Fusobacterium nucleatum* Ct value. *Fn, Fusobacterium nucleatum.*

Overall survival was analyzed using Kaplan–Meier curves and the log-rank test, as shown in Figure 4. CRC patients with high *Fusobacterium nucleatum* levels (Ct < 6.8) tended to have shorter overall survival compared with patients with low *F. nucleatum* levels (Ct > 6.8), although this difference was not statistically significant (*p* = 0.5) (Figure 4).

## 4 Discussion

*Fusobacterium nucleatum* has been associated with the development and progression of colorectal cancer (CRC) via multiple mechanisms. In recent years, a growing number of studies have investigated the association between *Fusobacterium nucleatum* and colorectal cancer (Kostic et al., 2012; Castellarin et al., 2012; Hashemi Goradel et al., 2019; McCoy et al., 2013). However, the precise mechanisms and interactions between *Fusobacterium nucleatum* and other elements of the tumor microenvironment (TME) in CRC development are still not well understood. In this study, we evaluated the abundance of *Fusobacterium nucleatum* (*Fn*) in colorectal cancer (CRC) tissues and its association with clinicopathological and molecular characteristics among CRC patients. Our findings demonstrated that *Fusobacterium nucleatum* was significantly more abundant in tumor tissues compared with adjacent normal mucosa, confirming its enrichment within the tumor microenvironment. These results are consistent with previous studies supporting a role for *F. nucleatum* in colorectal carcinogenesis (Rubinstein et al., 2013; McCoy et al., 2013; Zhang et al., 2018).

*F. nucleatum* was detected in 35.4% of CRC tissues compared to 19.5% of matched normal tissues, showing a statistically significant difference (*p* = 0.0005) (Figure 1). This differential abundance further supports the hypothesis that *F. nucleatum* actively involved in CRC pathogenesis (Castellarin et al., 2012; Viljoen et al., 2015; Rye et al., 2022).

The prevalence of *F. nucleatum* observed in this study falls within the average range (8.6–87%) reported in previous studies conducted in various countries (Mima et al., 2015; Suehiro et al., 2017; Wong et al., 2017; Tunsjø et al., 2019; Kim et al., 2020; Shariati et al., 2021). This variation can be attributed to multiple factors, including the use of different diagnostic techniques, the type of biological samples analyzed, and differences in sample quality (Suehiro et al., 2017; Li et al., 2016; Leung et al., 2019). In our previous studies we found *F. nucleatum* DNA in 37% of CRC biopsies tissue by comparing the frequency of *F. nucleatum* in the colorectal tumor specimens and matched normal tissue by quantitative PCR analysis (Kulmambetova et al., 2024). Additional factors contributing to the inconsistency of findings include variations in the population’s gut microbiome, dietary patterns, and geographical differences.

While *F. nucleatum* abundance showed no significant relationship with traditional clinicopathological factors including age, sex, tumor size, and TNM stage, some important correlations were observed. *F. nucleatum* levels were significantly increased in MSI-high tumors (*p* = 0.01), supporting prior evidence that this bacterium preferentially colonizes microsatellite-unstable cancers. Additionally, European nationality (p = 0.01), and high consumption of processed meat (*p* = 0.0009) were all positively correlated with *Fn*-high status. These associations suggest that host factors such as ethnicity and comorbidities, along with dietary habits, may influence microbial colonization patterns in CRC.

The dietary findings are particularly compelling. More recent studies have reported that the association between *Fusobacterium nucleatum* and colorectal cancer (CRC) appears to be stronger in Asian populations compared to those in Europe or North America (Nishijima et al., 2016; Huang et al., 2018; Janati et al., 2020). Patients with high processed meat intake showed significantly higher *F. nucleatum* levels, supporting existing evidence that Western-style diets rich in processed meats may promote a tumor microenvironment favorable to *F. nucleatum*. In contrast, previous literature suggests that diets high in fiber and plant-based foods are protective against *Fn*-positive CRC, likely due to their beneficial effects on gut microbiota composition. These dietary patterns play a significant role in shaping the composition and diversity of the colonic microbiota. Importantly, maintaining a balance between health-promoting and harmful microbial metabolites plays a critical role in modulating CRC risk (Zhang et al., 2018; Leng et al., 2018). Nonetheless, further research is needed to explore these associations in greater depth, taking into account a wider range of contributing factors.

The exact mechanisms by which the gut microbiota contributes to colorectal cancer development remain incompletely understood. Inflammation is a well-recognized risk factor that significantly contributes to both the initiation and progression of CRC (Gerhard Rogler et al., 2011). Disruptions in the composition of the gut microbiota—known as dysbiosis—can lead to immune imbalance and heightened production of pro-inflammatory mediators (Swidsinski et al., 2011). A number of studies have investigated the role of *Fusobacterium nucleatum* in colorectal tumorigenesis (Hashemi Goradel et al., 2019; McCoy et al., 2013), demonstrating that its presence in the gut stimulates the expression of tumor-associated cytokines and activates inflammatory pathways, largely through the action of its virulence factors.

Our analyses revealed a significant association between elevated *Fusobacterium nucleatum* levels and increased tumor expression of the pro-inflammatory cytokines *IL-6* and *IL-17* (both *p* = 0.02). These cytokines are key mediators of inflammation and tumor progression, and their elevated expression in *Fn*-high tissues points toward a possible mechanism by which *F. nucleatum* contributes to CRC development—through inflammation-driven carcinogenesis.

Interestingly, no significant associations were found between *F. nucleatum* levels and other cytokines, including IL-1*β*, IL-10, and TNF-*α*, indicating a more specific inflammatory profile associated with its presence.

*Fusobacterium nucleatum* has been reported to activate β-catenin signaling through two distinct mechanisms. The first involves the interaction between its adhesin FadA and host cell E-cadherin, which triggers a mechanism that facilitates bacterial invasion. The second mechanism operates through the TLR4/P-PAK1 signaling pathway, leading to an inflammatory response and upregulation of NF-κB target genes and pro-inflammatory cytokines. In addition to these mechanisms, *F. nucleatum* contributes to inflammation through its Fap2 protein, which binds to the Gal-GalNAc receptor (Rubinstein et al., 2013; Kostic et al., 2013). Toll-like receptors, particularly TLR2 and TLR4, are critical for recognizing *F. nucleatum* and mediating its pro-inflammatory effects, in part through the modulation of regulatory T cells (Tregs) (Jia et al., 2017). The findings (Rubinstein et al., 2013; Kostic et al., 2013) further support the role of *Fusobacterium nucleatum* in promoting a pro-inflammatory tumor microenvironment, thereby enhancing its oncogenic potential.

Based on these findings, we assessed the expression of mucosal inflammatory cytokines and examined their correlation with *Fusobacterium nucleatum* abundance in colorectal cancer tissues versus normal tissues. Our results demonstrated that *IL-6*, *IL-1β*, and *IL-10* were overexpressed in tumor tissues compared with normal mucosa. We also observed a significant positive correlation between high *F. nucleatum* levels and elevated *IL-17* expression in tumor tissues.

Interleukin-6 (*IL-6*) is a pro-inflammatory cytokine with pro-tumorigenic properties. It plays a central role in regulating multiple signaling pathways that influence cell survival, invasion, apoptosis, proliferation, angiogenesis, and metastasis. Elevated IL-6 expression has been widely reported in various malignancies, including colorectal cancers (Sethi et al., 2012; Heichler et al., 2020; Ke et al., 2020). Consistent with these findings, we observed significantly increased IL-6 levels in tumor tissue of colorectal cancer. However, some studies, such as that by Heikkilä et al. (2009), did not find a significant association between IL-6 levels and the risk of developing colorectal cancer (CRC), possibly due to limitations such as small sample size (Heikkilä et al., 2009). In contrast, a separate study involving 208 CRC patients across stages I–IV reported that serum IL-6 concentrations were significantly higher in individuals with stage III and IV disease compared to those with earlier-stage CRC (Belluco et al., 2000). Moreover, chronic inflammation and tissue injury, as observed in various gastrointestinal and hepatic disorders, maintain persistent IL-6/STAT3 activation that supports the proliferation of mutated epithelial cells and contributes to tumor initiation and progression (He and Karin, 2011).

Tumor necrosis factor-alpha (TNF-*α*), a pro-inflammatory cytokine secreted by both tumor and immune cells, similarly plays a crucial role in regulating multiple signaling pathways. Like IL-6, TNF-α contributes to tumorigenesis by promoting cell proliferation, angiogenesis, metastasis, and even participating in the early stages of tumor initiation (Lan et al., 2021). Natural killer (NK) cells can directly recognize *F. nucleatum* through its surface ligands and respond by releasing TNF-α, which in turn enhances IL-6 expression and secretion (Chaushu et al., 2012). Several studies on colorectal adenomas have also demonstrated a positive correlation between elevated *Fusobacterium nucleatum* levels and increased expression of inflammatory cytokines, including IL-6 and TNF-α (McCoy et al., 2013; Velsko et al., 2015). While McCoy et al. (2013) observed a positive trend between *Fusobacterium* species and IL-6 expression, the findings did not achieve statistical significance. Additional studies have reported that inflammatory markers such as IL-1β, IL-6, and TNF-α are strongly associated with *F. nucleatum* infection and are more highly expressed in colorectal cancers harboring this bacterium. These effects, however, were not detected in CRC tissues colonized by other bacterial species (Wu et al., 2019). Consequently, elevated circulating levels of *IL-6* and *TNF-α*—key markers of gastrointestinal inflammation—may serve as indicators of CRC progression potentially driven by *Fusobacterium nucleatum.* In our study, we did not find any correlation between *F. nucleatum* prevalence and TNF-α expression levels.

Interleukin-17 (IL-17), another cytokine analyzed in this study, is predominantly produced by Th17 lymphocytes and plays a central role in driving chronic inflammation. In addition to its pro-inflammatory function, IL-17 is a potent immunomodulator that can promote angiogenesis and support tumor growth (Kuen et al., 2020).

Our findings did not reveal a significant increase in IL-17 expression levels in CRC tissues compared to matched normal tissues. But we demonstrated positive significance correlation of expression level of IL-17with high abundance of *F. nucleatum* in tumor tissue. This finding is consistent with previous studies demonstrating the role of IL-17 in the development and progression of CRC (Feng et al., 2019; Lin et al., 2015). In hepatocellular carcinoma, particularly in alcohol-related liver disease, IL-17–mediated activation of hepatocytes and macrophages drives inflammation and tumor development, while pathway inhibition attenuates these effects (Ma et al., 2020).

In the context of chronic versus acute inflammation, IL-6 is typically associated with acute-phase responses, whereas IL-17 supports chronic mucosal inflammation. *F. nucleatum* may establish a chronic inflammatory milieu dominated by IL-17A rather than IL-6. In chronic inflammation, IL-17A primarily stimulates epithelial and stromal cells rather than immune cells, thereby shifting cytokine dynamics. IL-6 expression may also be downregulated through feedback loops when IL-17A is predominant. This pattern may reflect a dysregulated mucosal immune response in which *F. nucleatum* disrupts the normal balance to promote a tumor-supportive chronic inflammatory state (IL-17–dominant), while bypassing the IL-6–driven acute immune response that might otherwise trigger stronger cytotoxic activity. Thus, *Fusobacterium nucleatum* may steer the immune system toward a chronic, IL-17–dominant state, ultimately facilitating tumor survival and immune evasion.

Interleukin-10 (IL-10) is a cytokine with dual immunomodulatory roles. On one hand, its immunosuppressive activity—particularly on dendritic cells and macrophages—reduces antigen presentation, facilitating tumor immune evasion and impairing the maturation and differentiation of immune effector cells. On the other hand, IL-10 can exert a potential antitumor effect by inhibiting the NF-κB signaling pathway and thereby suppressing pro-inflammatory cytokine production (Li et al., 2020; Abtahi et al., 2017). Previous studies (Abtahi et al., 2017) have reported significantly lower serum IL-10 levels in colorectal cancer patients compared with healthy controls, which is consistent with our findings. However, their study also found that elevated IL-10 expression was linked to poorer prognosis in CRC patients. In contrast, other studies have observed increased IL-10 expression in CRC tissues relative to normal tissues (Li et al., 2019). These conflicting findings may reflect differences in disease stage, tumor microenvironment, or methodological approaches, underscoring the complex and context-dependent role of IL-10 in CRC. Thus, further investigation is needed to elucidate the mechanisms by which IL-10 may contribute to either tumor suppression or progression.

In colorectal cancer, the tumor microenvironment is deeply involved in both the initiation and advancement of the disease, with interleukin-1β (IL-1β) serving as a key mediator in several associated processes. IL-1β is markedly overexpressed in the intestinal mucosa of CRC patients compared to normal tissues. Moreover, lipopolysaccharide (LPS) has been shown to induce IL-1β production in neutrophils (Li et al., 2020). In the context of colitis-associated cancer (CAC), neutrophil-derived IL-1β stimulates intestinal mononuclear phagocytes (MPs) to produce IL-6, thereby promoting tumor growth. A newly proposed framework—the complement/neutrophil/IL-1β–myeloid cell/IL-17A axis—further clarifies the involvement of the complement system in colorectal cancer progression (Ning et al., 2015). Together, these findings underscore the strong connection between the TME and IL-1β activity, suggesting that therapeutic strategies targeting components of the TME could offer promising avenues for CRC treatment. While its overexpression promotes inflammation, tumor invasion, and metastasis, certain genetic polymorphisms in the IL1B gene (e.g., rs1143627, rs16944) have been linked to increased risk of cervical cancer, whereas others (rs3136558, rs1143630) may exert protective effects (Wang et al., 2019). With regard to lung cancer, certain genetic variants of the IL-1β gene (e.g., the G allele of rs1143633) are protective. These observations highlight the complex interplay of IL-1β gene polymorphisms, environmental factors, and cancer risk (Yin et al., 2023).

IL-6, IL-1β, IL-10, IL-17, and TNF-*α* were selected as key mediators of inflammation and immune regulation in colorectal cancer. These cytokines represent complementary aspects of the tumor microenvironment: pro-inflammatory and tumor-promoting signaling (IL-6, IL-1β, TNF-α), anti-inflammatory modulation (IL-10), and Th17-driven immune activity (IL-17). Their combined assessment enables a comprehensive evaluation of inflammatory balance and its potential association with *Fusobacterium nucleatum* and colorectal carcinogenesis (Li et al., 2020). Cytokine expression was quantified using droplet digital PCR with TaqMan probes, which enables absolute quantification without standard curves and provides high sensitivity and specificity. This approach ensures reliable detection of low-abundance transcripts and reproducible results even in partially degraded RNA samples, making it well suited for focused genes such as IL-6, IL-1β, IL-10, IL-17, and TNF-α.

While the Kaplan–Meier survival analysis showed a trend toward poorer overall survival in *F. nucleatum*-high patients, the difference was not statistically significant (*p* = 0.5). Although inconclusive, this trend warrants further investigation in larger cohorts to clarify whether *F. nucleatum* abundance can serve as a prognostic biomarker in CRC.

Lastly, the stage-wise analysis of *F. nucleatum* abundance in both tumor and distant mucosa did not reveal a clear progression-related trend. Although *F. nucleatum* levels were elevated in stages II and III compared to normal mucosa, there were no significant differences across tumor stages, suggesting that its presence is not strictly stage-dependent but may be more associated with tumor subtype or microenvironmental factors.

Despite the robust design of this study, several limitations must be acknowledged. The sample size, while moderate, may not have been sufficient to detect weaker associations or survival differences. Additionally, although relative quantification methods were used to measure *F. nucleatum*, absolute quantification could offer more precision.

Future studies should focus on longitudinal designs to assess whether *F. nucleatum* colonization precedes tumor development or reflects a consequence of tumorigenesis. Further exploration of its interaction with host immunity, microbiota composition, and diet will be critical in understanding its role as a potential biomarker or therapeutic target.

Our findings reinforce the significant enrichment of *F. nucleatum* in CRC tissues and its association with specific clinical, dietary, and molecular features. The observed correlations with MSI-high status, pro-inflammatory cytokine expression, and dietary factors suggest a multifaceted role for *F. nucleatum* in colorectal cancer pathogenesis. These findings add to the accumulating evidence supporting for the integration of microbiome analysis into CRC research and clinical practice. The present findings shed light on the role of *F. nucleatum* and its potential link to elevated gene expression in carcinogenesis, highlighting its critical involvement in CRC progression. The data provide substantial evidence for the pathogenic contribution of *F. nucleatum* in CRC, suggesting new opportunities to target the microbiota in order to improve prognosis and prevent disease development. Moreover, given its impact on inflammatory mediators and their expression, *F. nucleatum* and related factors may serve as promising biomarkers for cancer diagnosis.

## Data Availability

The original contributions presented in the study are included in the article/Supplementary material, further inquiries can be directed to the corresponding author/s.

## Footnotes

^1^ https://www.r-project.org
