# Supplementary material for: Investigation of the relationship between Fusobacterium nucleatum presence and inflammatory mediator expression in Kazakhstani patients with colorectal cancer
Source: Front Microbiol. 2025 Nov 5;16:1699745. doi: 10.3389/fmicb.2025.1699745 (PMC12626992; doi:10.3389/fmicb.2025.1699745)
Supplement: Supplementary file 1 [file Data_Sheet_1.docx]

Supplementary Material

# Supplementary Tables

## Table 1 Clinicopathological and molecular characteristics of paired colorectal adenocarcinoma and matched non-tumor tissue samples

| Parameter | Tumor tissue (n = 113) | Matched non-tumor tissue (n = 113) | p-value |
| --- | --- | --- | --- |
| Source | Colorectal adenocarcinoma | Adjacent normal mucosa (≥10 cm from tumor margin) | – |
| Histology | Adenocarcinoma | Normal colorectal epithelium | – |
| Tumor differentiation | Well: 1 (0.9%) Moderate: 49 (43.4%) Poor: 63 (55.7%) | – | – |
| TNM stage | I–II: 60 (54%) III–IV: 51 (46%) | – | – |
| Tumor location | Right: 35 (31%) Left: 78 (69%) | Same patient, same region | – |
| Tumor size (cm, mean ± SD) | 4.7 ± 2.5 | – | – |
| Age (years, mean ± SD) | 61.7 ± 10.9 | 61.7 ± 10.9 | – |
| Sex (M/F) | 50 / 63 | 50 / 63 | – |
| *F. nucleatum* detection, n (%) | 40 (35.4%) | 22 (19.5%) | <0.001 |

Notes:

- Matched non-tumor tissue — morphologically normal mucosa taken from the same patient (≥10 cm from the tumor margin).
- Statistical analysis — paired T test.
- p-values indicate comparison between tumor and matched non-tumor tissues.

Table 2 Average Ct values of SLCO2A1 in tumor vs. normal tissues in colorectal cancer patients

| ID | Ct value of SLCO2A1 in CT | Ct value of SLCO2A1 in NT | pSt |
| --- | --- | --- | --- |
| 1 | 23.24 | 21.96 | 0 |
| 2 | 20.82 | 19.90 | 3 |
| 3 | 22.77 | 21.77 | 3 |
| 4 | 20.18 | 19.88 | 3 |
| 5 | 23.45 | 21.79 | 2 |
| 6 | 18.25 | 19.21 | 3 |
| 7 | 22.04 | 21.99 | 2 |
| 8 | 21.40 | 21.25 | 3 |
| 9 | 22.83 | 21.53 | 2 |
| 10 | 23.21 | 21.32 | 3 |
| 11 | 20.49 | 22.12 | 2 |
| 12 | 21.21 | 21.61 | 1 |
| 13 | 21.56 | 22.16 | 3 |
| 14 | 22.11 | 21.97 | 1 |
| 15 | 21.26 | 21.91 | 3 |
| 16 | 22.59 | 21.03 | 3 |
| 17 | 23.09 | 21.86 | 2 |
| 18 | 21.68 | 21.41 | 3 |
| 19 | 20.87 | 21.21 | 2 |
| 20 | 22.69 | 21.83 | 3 |
| 21 | 22.72 | 21.60 | 3 |
| 22 | 21.95 | 23.72 | 3 |
| 23 | 22.74 | 22.48 | 3 |
| 24 | 22.35 | 21.88 | 3 |
| 25 | 22.72 | 22.34 | 2 |
| 26 | 22.71 | 23.00 | 3 |
| 27 | 22.59 | 23.08 | 2 |
| 28 | 22.31 | 23.93 | 3 |
| 29 | 21.63 | 22.21 | 2 |
| 30 | 22.79 | 22.92 | 3 |
| 31 | 23.09 | 21.06 | 2 |
| 32 | 21.44 | 21.30 | 2 |
| 33 | 22.02 | 22.19 | 3 |
| 34 | 22.79 | 23.88 | 1 |
| 35 | 21.32 | 22.12 | 2 |
| 36 | 22.54 | 21.26 | 2 |
| 37 | 23.22 | 21.51 | 1 |
| 38 | 21.29 | 22.07 | 2 |
| 39 | 22.43 | 21.98 | 1 |
| 40 | 25.40 | 21.92 | 1 |
| 41 | 22.47 | 23.29 | 2 |
| 42 | 21.55 | 23.70 | 2 |
| 43 | 22.24 | 22.04 | 3 |
| 44 | 21.73 | 21.74 | 2 |
| 45 | 20.25 | 21.20 | 1 |
| 46 | 21.96 | 21.75 | 2 |
| 47 | 22.99 | 21.35 | 3 |
| 48 | 21.69 | 22.18 | 2 |
| 50 | 22.14 | 21.64 | 3 |
| 51 | 21.92 | 21.76 | 3 |
| 52 | 21.45 | 22.19 | 2 |
| 53 | 23.43 | 22.57 | 3 |
| 54 | 23.16 | 22.94 | 2 |
| 55 | 23.77 | 25.04 | 2 |
| 56 | 20.86 | 24.23 | 3 |
| 57 | 25.08 | 23.16 | 2 |
| 58 | 21.78 | 21.75 | 2 |
| 59 | 21.69 | 22.34 | 2 |
| 60 | 23.06 | 24.29 | 3 |
| 61 | 22.14 | 21.71 | 3 |
| 62 | 22.04 | 23.08 | 3 |
| 63 | 21.21 | 20.95 | 1 |
| 64 | 23.71 | 25.44 | 1 |
| 65 | 22.01 | 21.89 | 1 |
| 66 | 24.40 | 23.23 | 3 |
| 67 | 22.14 | 22.68 | 3 |
| 68 | 22.00 | 22.38 | 3 |
| 69 | 23.28 | 21.63 | 2 |
| 70 | 24.48 | 24.39 | 3 |
| 71 | 21.88 | 21.16 | 3 |
| 72 | 21.96 | 22.09 | 2 |
| 73 | 21.81 | 22.71 | 1 |
| 74 | 24.55 | 23.04 | 2 |
| 75 | 25.80 | 23.96 | 2 |
| 76 | 23.71 | 24.05 | 3 |
| 77 | 23.72 | 22.28 | 3 |
| 78 | 23.34 | 23.65 | 2 |
| 80 | 22.88 | 22.57 | 2 |
| 81 | 26.26 | 21.13 | 2 |
| 82 | 21.81 | 23.48 | 4 |
| 83 | 21.86 | 25.64 | 3 |
| 84 | 22.66 | 23.75 | 2 |
| 85 | 23.25 | 23.02 | 2 |
| 86 | 21.15 | 21.32 | 2 |
| 87 | 21.94 | 22.16 | 3 |
| 88 | 22.81 | 19.97 | 2 |
| 89 | 23.88 | 21.41 | 1 |
| 90 | 22.13 | 21.25 | 2 |
| 91 | 21.14 | 21.16 | 3 |
| 92 | 22.16 | 23.14 | 2 |
| 93 | 22.16 | 22.62 | 3 |
| 94 | 22.20 | 22.26 | 2 |
| 95 | 20.75 | 21.74 | 3 |
| 96 | 22.14 | 22.08 | 3 |
| 97 | 22.08 | 21.32 | 2 |
| 98 | 20.66 | 22.21 | 2 |
| 99 | 22.31 | 21.35 | 3 |
| 100 | 22.22 | 21.58 | 3 |
| 101 | 24.30 | 23.50 | 3 |
| 102 | 22.70 | 22.71 | 3 |
| 103 | 21.43 | 22.93 | 1 |
| 104 | 22.37 | 23.78 | 0 |
| 105 | 22.75 | 23.07 | 3 |
| 106 | 23.42 | 26.55 | 3 |
| 107 | 24.13 | 25.35 | 3 |
| 108 | 26.43 | 27.75 | 3 |
| 109 | 22.75 | 25.16 | 2 |
| 110 | 24.81 | 22.64 | 2 |
| 111 | 23.90 | 23.26 | 2 |
| 112 | 23.74 | 25.83 | 2 |
| 113 | 22.99 | 23.37 | 3 |
| 114 | 22.67 | 22.78 | 2 |

**Notes:**

- *Ct value of SLCO2A1 in CT* – average Ct value of *SLCO2A1* in tumor tissue.
- *Ct value of SLCO2A1 in* *NT* – average Ct value of *SLCO2A1* in matched normal tissue.
- *pSt* – tumor pathological stage (0 = precancerous lesion; 1 = Stage I; 2 = Stage II; 3 = Stage III, according to TNM classification).

## Supplementary Figures


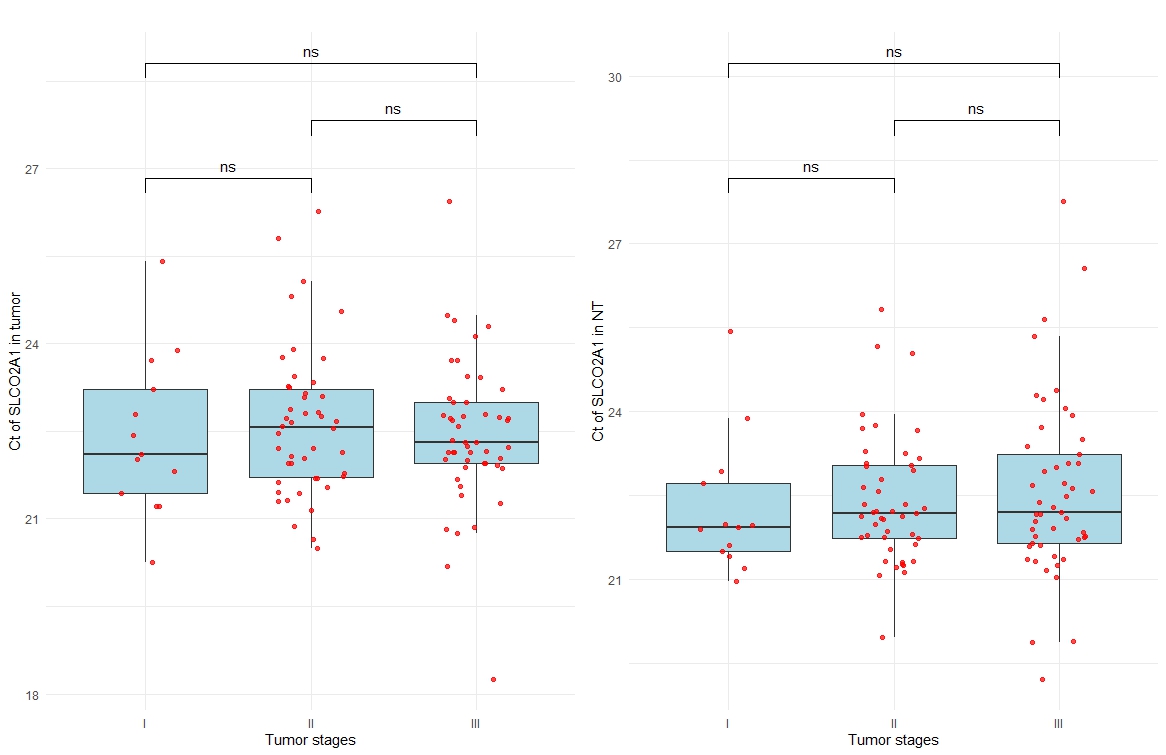


**Supplementary Figure 1.** Comparison of SLCO2A1 Ct values across tumor stages in colorectal cancer patients.

Boxplots showing Ct values of SLCO2A1 in tumor (left) and normal (right) tissues across different tumor stages (I–III) in colorectal cancer patients. Red points represent individual data values. Statistical analysis revealed no significant differences between stages (ns).
